# A systematic review of human pathogens carried by the housefly (*Musca domestica* L.)

**DOI:** 10.1186/s12889-018-5934-3

**Published:** 2018-08-22

**Authors:** Faham Khamesipour, Kamran Bagheri Lankarani, Behnam Honarvar, Tebit Emmanuel Kwenti

**Affiliations:** 1 Health Policy Research Center, Institute of Health, Shiraz University of Medical Science, Shiraz, Iran; 2 Student Research Committee, Shiraz University of Medical Sciences, Shiraz, Iran; 3 Department of Microbiology and Parasitology, University of Buea, Buea, Cameroon; 4 Department of Medical Laboratory Science, Faculty of Health science, University of Buea, Buea, Southwest Region Cameroon

**Keywords:** Bacteria, Fungi, House fly, House fly control, Mechanical transmission, Parasites, Pathogens, Viruses

## Abstract

**Background:**

The synanthropic house fly, *Musca domestica* (Diptera: Muscidae), is a mechanical vector of pathogens (bacteria, fungi, viruses, and parasites), some of which cause serious diseases in humans and domestic animals. In the present study, a systematic review was done on the types and prevalence of human pathogens carried by the house fly.

**Methods:**

Major health-related electronic databases including PubMed, PubMed Central, Google Scholar, and Science Direct were searched (Last update 31/11/2017) for relevant literature on pathogens that have been isolated from the house fly.

**Results:**

Of the 1718 titles produced by bibliographic search, 99 were included in the review. Among the titles included, 69, 15, 3, 4, 1 and 7 described bacterial, fungi, bacteria+fungi, parasites, parasite+bacteria, and viral pathogens, respectively. Most of the house flies were captured in/around human habitation and animal farms. Pathogens were frequently isolated from body surfaces of the flies. Over 130 pathogens, predominantly bacteria (including some serious and life-threatening species) were identified from the house flies. Numerous publications also reported antimicrobial resistant bacteria and fungi isolated from house flies.

**Conclusions:**

This review showed that house flies carry a large number of pathogens which can cause serious infections in humans and animals. More studies are needed to identify new pathogens carried by the house fly.

**Electronic supplementary material:**

The online version of this article (10.1186/s12889-018-5934-3) contains supplementary material, which is available to authorized users.

## Background

The house fly, *Musca domestica* L. (Diptera: Muscidae), is the most common and widespread species of fly in the world. It is said to have originated from the savannahs of Central Asia and spread throughout the world, and can be found in both rural and urban areas of tropical and temperate climates [1, 2]. The house fly belongs to a group of flies often referred to as “filth flies”; the other members belong to the families Calliphoridae and Fanniidae [3]. The house fly has been in existence since the origin of human life [4] and well adapted to life in human habitations [5]. *M. domestica* is an eusynanthropic, endophilic species, i.e. it lives closely in association with humans and is able to complete its entire lifecycle within habitations of humans and domestic animals [6]. House flies are often found in abundance in areas of human activities such as hospitals, food markets, slaughter houses, food centers or restaurants, poultry and livestock farms where they constitute a nuisance to humans, poultry, livestock and other farm animals, and also act as potential vector of diseases [7].

The house fly is known to carry pathogens that can cause serious and life-threatening diseases in humans and animals. Over 100 pathogens including bacteria, viruses, fungi and parasites (protozoans and metazoans) have been associated with the insect [8, 9]. Molecular analysis revealed that house flies carry very diverse groups of microorganisms [10]. Evidence supporting the role of the house fly in transmission of diseases are mostly circumstantial, with the strongest evidence pointing to the correlation between the rise in incidence of diarrhoea and an increase in the fly population [11–14].

The characteristics of the pathogens carried by house flies depend on the area where the insect is collected; house flies captured from the hospital environment or animal farms (where there is extensive use of antibiotics as growth promoters) commonly carry antimicrobial resistant bacteria and fungi [9, 15–20]. More so, house flies presenting in the hospital environment may also be associated with the transmission of nosocomial infections [9, 21].

House fly causes mechanical transmission of pathogens, which is the most widely recognised mechanism [22–24]. This occurs when pathogens are transmitted from one vertebrate hosts to another without amplification or development of the organism within the vector [22]. House flies usually feed and reproduce in feces, animal manure, carrion and other decaying organic substances, and thus live in intimate association with various microorganisms including human pathogens, which may stick to body surfaces of the fly. The constant back and forth movement of house flies between their breeding sites and human dwellings can lead to the transmission of pathogens to humans and animals.

Currently, there is no systematic review on the pathogens carried by the house fly. The aim of this systematic review was to identify the types and prevalence of human pathogens carried by the house fly.

## Methods

For this systematic review, we did a literature search to identify scientific articles reporting pathogens (bacteria, viruses, fungi and parasites) that has been isolated from the house fly (*Musca domestica*). The current study conforms to the Preferred Reporting Items for Systematic reviews and Meta-analyses (PRISMA) guidelines [25] (Additional file 1).

### Search strategy and selection criteria

Relevant studies were searched in health-related electronic databases including PubMed, PubMed Central, Google Scholar and Science Direct using the keywords: House fly OR *Musca domestica* OR Pathogens OR bacteria OR fungi OR parasites OR viruses.

**Fig. 1:**
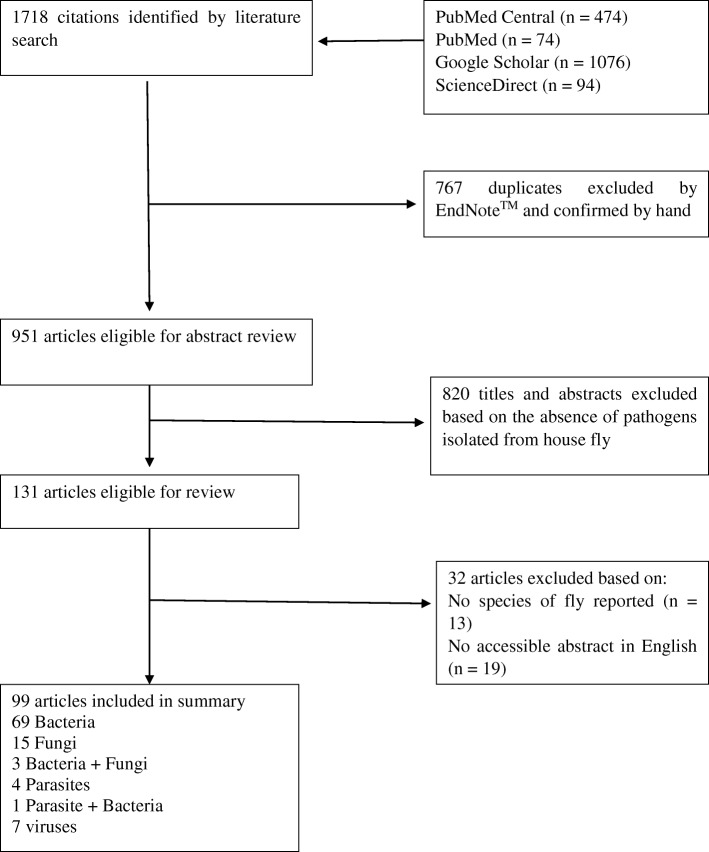
Flowchart of the selection process for publications included in this review

The search was limited to the studies published in English or containing at least an English abstract until November 2017. Subsequently, the titles and abstracts of the selected articles were examined by 2 reviewers, independently (parallel method) to identify articles reporting pathogens isolated from the house fly. When there was any discrepancy in their report, a third reviewer was invited to resolve the issue. Relevant papers were also manually cross checked in order to identify further references. In the selected articles, the following data were extracted by the first reviewer and checked by the second reviewer. The data included type and species of pathogen isolated, stage of house fly from which pathogen was isolated, frequency of occurrence of pathogen, method used in isolation of pathogen, type of study (field or experimental), site of the house fly from where the pathogen was isolated, nature of pathogen isolated (whether the pathogen was carrying genes that were resistant to antimicrobials or not), and location of capture of the house fly (human residents, animal farms, markets/shops, hospitals etc.). Excluded articles were those reporting pathogens isolated from flies in general without specifying the fly species. The selection process is detailed in Fig.1.

### Risk of bias in individual studies

Level of risk of bias for the study was likely to be high mainly because of differences in study and the methods used to isolate pathogens from the house fly. Most of the studies were not designed to isolate all the types of pathogens. Moreover, studies using molecular methods (PCR and/or sequences) yielded more pathogens compared to studies using standard cultural methods.

## Results

Figure 1 (PRISMA flowchart) provides a four-phase study selection process in the present systematic review study. A total of 1718 studies were identified in the initial search. After the title and abstract screening, 131 full- text articles were retrieved. Of these, a final 99 articles were identified for this review [2–24, 26–93]

Seventy-three 73 (73.73%) of the works described bacterial pathogens (Table 1), 18 (18.18%) fungi (Table 2), 5 (5.05%) parasites (Table 3) and 7 (7.07%) described viruses. The selected studies were done in 21 countries and the study period covered the years 1970–2017. Sixty-eight of the studies were field studies (performed on house flies caught in the wild) (68.69%) while 31 were experimental studies (performed in the laboratory) (31.31%). Of the 68 field studies, 12 described pathogens isolated from house flies caught in the wild in Europe, 16 in the Middle East, 15 in Africa, 13 in USA, 10 in Asia, and 2 in South America. Twenty studies (28.88%) reported on house flies that were caught from within human habitation, 28 (28.28%) from animal farms (including poultry, dairy and piggery farms), 10 (10.10%) from the surroundings, 10 (10.10%) from food centers (including cafeteria, restaurants), 7 (7.07%) from markets or shops, 14 (14.14) from hospitals, 7 (7.07%) from dump sites or sanitary landfills while 4 (4.04%) were from gardens or farms.

**Table 1:** Bacteria species that have been isolated from house flies, including the site of isolation, the frequencies and their distribution

Bacteria genera	Species	Medical and/or veterinary importance	Geographical occurrence	Site of specimen collection	Host stage infected	Prevalence	Lab or field study	Site of isolation	References
*Helicobacter*	*H. pylori*	Medical	Worldwide	Laboratory reared	Adult	–	Lab	External surfaces/internal organs	[70–72]
*Campylobacter*	*C. jejuni*	Medical and veterinary	Worldwide	Poultry, piggery	Adult/larvae	6.2%	Field/ Lab	External surfaces/internal organs	[73, 74, 87, 90]
	*C. coli*	Medical and veterinary	Worldwide	Poultry, piggery	Adult	90.1%	Field	External surfaces/internal organs	[74]
	Others	Medical and veterinary	Worldwide	Restaurant, refuse dumps, barbecue shops, fruits and food vendors, markets, poultries (broiler farms)	Adult	–	Field	External surfaces/internal organs	[38, 75]
*Salmonella*	*S. typhimurium*	Medical	Worldwide	Laboratory experiment	Adult	–	Lab		[37]
	*S. enterica serovar Enteritidis*	Medical	Worldwide	Poultry, dumpsters	Adult/larvae	6–70%	Lab/field	External surfaces/internal organs	[23, 28, 39]
	Others	Medical	Worldwide	Restaurant, refuse dumps, barbecue shops, fruits and food vendors, markets, fish vendors, human habitation	Adult	11.8–66.67%	Field	External surfaces/internal organs	[25, 27, 29, 70–72, 91]
*Escherichia*	*E. coli*	Medical	Worldwide	Human habitation, cafeteria and food centers, hospitals, open fields, poultry farms, slaughter houses, cattle farms, animal hospitals	Adult/larvae	10.5–76.3%	Field/lab	External surfaces/internal organs	[2–24, 29–38, 40, 43–46, 48, 89, 92]
*Bacillus*	*B. anthrax*	Medical and veterinary	Worldwide	Laboratory reared	Adult	–	Lab	External surfaces/Internal organs	[76]
	*B. megatarium*	non	Worldwide	Cafeteria and food centers	Adult	50%	Field	External surfaces/Internal organs	[7]
	*B. sphaericus*	Medical	Worldwide	Cafeteria and food centers	Adult	50%	Field	External surfaces/Internal organs	[7]
	*B. cereus*	Medical	Worldwide	Fresh fish	Larvae	–	Field	External surfaces/Internal organs	[46, 53]
	*B. alvei*	Medical	Worldwide	Cafeteria and food centers	Adult	50%	Field	External surfaces/Internal organs	[7]
	*B. pumilus*	Medical	Worldwide		Adult	–	Field	External surfaces/Internal organs	[46]
	*B. thuringiensis*	non	Worldwide		Adult	–	Field	External surfaces/Internal organs	[46]
	Others	Medical	Worldwide	Dairy farms, hospitals, slaughter houses, fruit and food centers	Adult	31.1%	Field	External surfaces	[10, 20, 44, 61, 89]
*Staphylococcus*	*S. aureus*	Medical	Worldwide	Human habitation, fresh fish	Adult/ larvae	26.9%	Field	External surfaces/Internal organs	[40, 45, 53, 77, 92]
	*S. epidermidis*	Medical	Worldwide	Human habitation	Adult	–	Field	External surfaces/Internal organs	[40]
	*S. sciuri*	Medical/veterinary	Worldwide	Dumpsters of restaurants	Adult	–	Field	External surfaces	[46]
	*S. saprophyticus*	Medical	Worldwide	Dumpsters of restaurants	Adult	–	Field	External surfaces	[46]
	*S. xylosus*	Medical	Worldwide	Dumpsters of restaurants	Adult	–	Field	External surfaces	[46]
	Others	Medical/veterinary	Worldwide	Poutry, animal farms, garden, garbage/dump areas, restaurants/cafeteria, markets, human habitation, hospitals	Adult	22.9–28.2%	Field	External surfaces/Internal organs	[10, 20, 48, 78, 79, 89, 91]
*Enterococcus*	*E. faecalis*	Medical	Worldwide	Restaurants, piggery farms	Adult	55.5–88.2%	Field/lab	External surfaces/Internal organs	[21, 49, 50]
	*E. faecium*	Medical	Worldwide	Restaurants, piggery farms	Adult	6.8–12.8%	Field/lab	External surfaces/Internal organs	[49, 50]
	*E. casseliflavus*	Medical	Worldwide	Restaurants, piggery farms	Adult	4.9–6.7%	Field/lab	External surfaces/Internal organs	[49, 50]
	*E. hirae*	Medical/veterinary	Worldwide	piggery farms	Adult	12.8%	Field/lab	External surfaces/Internal organs	[50]
*Aeromonas*	*A. caviae*	Medical	Worldwide	Hospitals, streets, slaughter houses (abattoir)	Adult	39–78%	Field	Internal organs	[48, 80, 81]
	*A hydrophila*	Medical	Worldwide	Open field	Adult	–	Field	Internal organs	[82]
	Others	Medical	Worldwide	Poutry, animal farms, garden, garbage/dump areas, restaurants/cafeteria, markets, human habitation, hospitals	Adult	–	Field	Internal organs	[79]
*Shigella*	*S. sonnei*	Medical	Worldwide	Hospitals, streets, slaughter houses	Adult	–	Field	Internal organs	[48]
	*S. dysenteriae*	Medical	Worldwide	Dumpsters of restaurants	Adult	–	Field	Internal organs	[46]
	Others	Medical	Worldwide	Poultry, animal farms, garden, garbage/dump areas, restaurants/cafeteria, markets, human habitation, hospitals	Adult	4.8–66.67%	field	External surfaces/ internal organs	[11, 13, 29, 38, 40, 43, 79, 91]
*Klebsiella*	*K. pneumoniae*	Medical	Worldwide	Hospitals, human habitation, slaughter houses	Adult	11.3–82%	field	External surfaces/ internal organs	[15, 47, 82]
	*K. oxytoca*	Medical		Open field	Adult	–	Field	External surfaces	[83]
	Others	Medical		Poutry, animal farms,garden, garbage/dump areas, restaurants/cafeteria, markets, human habitation, hospitals, slaughter houses, open field	Adult	–	Field	External surfaces	[40, 44, 48, 61, 79, 89]
*Pseudomonas*	*P. aeruginosa*	Medical	Worldwide	Dump sites, slaughter houses, open field, human habitation, fresh fish, hospitals	Adult/larvae	37%	field	External surfaces/internal organs	[15, 19, 53, 61, 84]
	Others	Medical	Worldwide	Hospitals, streets, slaughter houses,	Adult	21.8%	Field	External surfaces/internal organs	[44, 48, 92]
*Proteus*	*P. mirabilis*	Medical	Worldwide	Slaughter houses, hospitals	Adult	29.1%	field	External surfaces/internal organs	[15]
	*P. vulgaris*	Medical	Worldwide	Human habitation	Adult	–	Field	External surfaces/internal organs	[40]
	*Proteus sp.*	Medical	Worldwide	Slaughter houses, dump sites, open fields, human habitations	Adult	14.8%	Field	External surfaces/internal organs	[61, 89, 92]
*Citrobacter*	*C. freundi*	Medical	Worldwide	Cafeteria and food centers, Slaughter houses, hospitals	Adult	28.4%	field	External surfaces/internal organs	[7, 15]
*Chronobacter*	*C. turicensis,*	Medical	Worldwide	Poultry, dumpsters	Adult/larvae	14%	Field/lab	External surfaces/internal organs	[23, 28]
	*C. universalis*	Medical	Worldwide	Poultry, dumpsters	Adult/larvae	–	Field/lab	External surfaces/internal organs	[23, 28]
	*C. sakazakii*	Medical	Worldwide	Poultry, dumpsters	Adult/larvae	–	Field/lab	External surfaces/internal organs	[23, 28, 46]
*Listeria*	*L. monocytogenes*	Medical	Worldwide	Poultry, dumpsters	Adult/larvae	3–49.4%	Field/lab	External surfaces/internal organs	[23, 28, 54]
	Others	Medical/veterinary	Worldwide	Animal farms	Adult	–	Field	External surfaces/internal organs	[78]
*Streptococcus*	*S. pyogenes*	medical	Worldwide	Fresh fish, human habitation	Adult/larvae	–	Field	External surfaces/internal organs	[40, 53]
	*S. faecalis*	Medical	Worldwide	Fresh fish	Larvae	–	Field	External surfaces/internal organs	[53]
	Others	Medical	Worldwide	Hospitals, slaughter houses, streets, dump sites, open fields, human habitation	Adult	66.67%	Field	External surfaces/internal organs	[48, 61, 89, 91]
*Alternaria*	*Alternaria spp.*	Medical	Worldwide	Fresh fish, Human habitation	Larvae	1.4–6%	Field		[15, 53, 55]
*Serratia*	*Serratia spp.*	Medical	worldwide	Human habitation	Adult	–	Field	Internal organs	[40]
*Enterobacter*	*Enterobacter spp.*	Medical	Worldwide	Human habitation	Adult	–	Field	Internal organs	[40, 89]
*Edwardsiella*	*Edwardsiella spp.*	Medical	Worldwide	Poultry	Adult	–	field	External surfaces/internal organs	[43]
*Providencia*	*Providencia spp.*	Medical	Worldwide	Poultry, animal farms, garden, garbage/dump areas, restaurants/cafeteria, markets, human habitation, hospitals, slaughter houses, open field	Adult	–	Field	External surfaces/ internal organs	[43, 79]
*Vibrio*	*Vibrio cholera* non O1	Medical	Worldwide	Human habitation	Adult	45.7%	Field	Internal organs	[29]
	Others	Medical / veterinary	Worldwide	Animal farms	Adult	–	Field	External surfaces/ internal organs	[78]
*Morganella*	*M. morgana*	Medical	Worldwide	Poutry, animal farms,garden, garbage/dump areas, restaurants/cafeteria, markets, human habitation, hospitals, slaughter houses, open field	Adult	16.67%	Field	External surfaces/Internal organs	[7, 79]
*Clostridium*	*Clostridium spp.*	Medical	Worldwide	Dairy farms	Adult	–	Field	Internal organs	[10]
*Corynebacterium*	*Corynebacterium spp.*	Medical	Worldwide	Dairy farms	Adult	–	Field	Internal organs	[10]
*Lactobacillus*	*Lactobacillus spp.*	Medical	Worldwide	Dairy farms	Adult	–	Field	Internal organs	[10]
*Yersinia*	*Y. enterocolitica*	Medical	Worldwide	Hospitals, streets, slaughter houses	Adult	–	Field	Internal organs	[48]
*Burkholderia*	*B. pseudomallei*	Medical	Worldwide	Open field	Adult	–	Field	Internal organs	[83]
*Acinetobacter*	*A. baumanni*	Medical	worldwide	Poultry, dumpsters	Adult	–	Field	Internal organs	[46, 89]
*Methylobacterium*	*M. persicinum*	Medical	Worldwide	Poultry, dumpsters	Adult	–	Field	Internal organs	[46]
*Micrococcus*	*Micrococcus sp.*	Medical	Worldwide	Garbage/dump areas, poultry, restaurants	Adult	–	Field	External surfaces/Internal organs	[89]

**Table 2:** Fungi species that have been isolated from house flies, including the site of isolation, the frequencies and their distribution

Fungi genera	Species	Medical, veterinary or agricultural importance	Geographical occurrence	Site of specimen collection	Host stage infected	Prevalence	Lab or field study	Site of isolation	References
*Cladosporum*	*C. cladosporoides*	Medical	Worldwide	Cattles diagnosed with bovine ringworm, animal pens, dump sites	Adult/larvae	4.7–85%	Field/lab	External surfaces	[56, 57]
	*Others*	Medical	Worldwide	Human habitation	Larvae	0.2	Field	External surfaces	[53, 55]
*Penicillium*	*P. axalicum*	Medical	Worldwide	Fresh fish	Larvae	–	Field	External surfaces	[53]
	*P. corylophilum*	Medical	Worldwide	Animal pens, dump sites	Larvae	–	Field	External surfaces	[57]
	*P. fellutanum*	Medical	Worldwide	Animal pens, dump sites	Larvae	11.9%	Field	External surfaces	[57]
	*P. verrucosum*	Medical	Worldwide	Human habitation	Adult	–	Field	External surfaces	[60]
	*P. aurantiogriseum*	Medical	Worldwide	Human habitation	Adult	–	Field	External surfaces	[60]
	Others	Medical	Worldwide	Human habitation, Cattles diagnosed with bovine ringworm, hospitals, slaughter houses	adult	3.4–21%	Field/lab	External surfaces	[55, 56, 58, 59]
*Aspergillus*	*A. flavus*	Medical	Worldwide	Animal pens, dump sites, poultry farms, dairy, piggery, slaughter houses, open field	Adult/larvae	23.8%	Field	External surfaces	[52, 57, 60]
	*A niger*	Medical	Worldwide	Animal pens, dump sites	Adult/larvae	14.4–85.71%	Field	External surfaces	[7, 57]
	*A fumigatus*	Medical	Worldwide	Cafeteria and food centers	Adult	85.71	Field	External surfaces	[7]
	*A tamari*	Medical	Worldwide	Fresh fish	Larvae	–	Field	External surfaces	[53]
	*A parasiticus*	Medical	Worldwide	Human habitation	Adult	–	Field	External surfaces	[60]
	Others	Medical	Worldwide	Human habitation, Cattles diagnosed with bovine ringworm, hospitals, slaughter houses	Adult	2.8–67.4%	Field/lab	External surfaces	[55, 56, 58, 59]
*Beauveria*	*B. bassiana*	Medical	Worldwide	Poultry farms, dairy, piggery, open field, slaughter houses	Adult	–	Field	External surfaces	[52]
*Mucor*	*M. cirinelloides*	Medical	Worldwide	Cafeteria and food centers	Adult	–	Field	External surfaces	[7]
	*Others*	Medical	Worldwide	Human habitation	Adult	2%	Field	External surfaces	[55]
*Alternaria*	*A. alternata*	Medical	Worldwide	Animal pens, dump sites	Adult/larvae	1.4–11.9%	Field	External surfaces	[53, 55, 57, 58]
*Fusarium*	*F. oxysporum*	Medical	Worldwide	Fresh fish	Larvae	–	Field	External surfaces	[53]
	*F. verticilliodes*	Medical	Worldwide	Human habitation	Adult	–	Field	External surfaces	[60]
	*F. proliferatum*	Medical	Worldwide	Human habitation	Adult	–	Field	External surfaces	[60]
	Others	Medical	Worldwide	Animal pens, dump sites, Human habitation, hospitals, slaughter houses	Larvae	4.7–17%	Field	External surfaces	[55, 57, 58]
*Curvularia*	*C. brachyspora*	Medical	Worldwide	Animal pens, dump sites	Adult	2.4%	Field	External surfaces	[57]
*Mycelia*	*M. sterilia*	Medical	Worldwide	Animal pens, dump sites	Adult	2.4%	Field	External surfaces	[57]
*Candida*	*C. albicans*	Medical	Worldwide	Pig pen, Human habitation	Adult	44.6%	Field	External surfaces	[51, 54]
	*C. glabrata*	Medical	Worldwide	Human habitation	Adult	23%	Field	External surfaces	[51]
	*C. krusei*	Medical	Worldwide	Human habitation	Adult	19.6%	Field	External surfaces	[51]
	*C. tropicalis*	Medical	Worldwide	Pig pen, Human habitation	Adult	7.4%	Field	External surfaces	[51, 54]
	*C. dubliniensis*	Medical	Worldwide	Human habitation	Adult	3.6%	Field	External surfaces	[51]
	*C. parapsilisis*	Medical	Worldwide	Human habitation	Adult	1.8%	Field	External surfaces	[51]
	Others	Medical	Worldwide	Human habitation	Adult	10.5%	Field	External surfaces	[59]
*Microsporum*	*M. canis*	Veterinary	Worldwide	Laboratory experiment	Adult/larvae	–	Field	External surfaces/internal organs	[85]
	*M. gypseum*	Medical	Worldwide	Hospitals, slaughter houses	Adult	–	Field	External surfaces	[58]
*Chrysosporium*	*Chrysosporium spp.*	Medical	Worldwide	Human habitation	Adult	2%	Field	External surfaces	[55]
*Curvalaria*	*Curvalaria spp.*	Agricultural	Worldwide	Human habitation	Adult	0.4%	Field	External surfaces	[55]
*Epicoccum*	*Epicoccum spp.*	Medical	Worldwide	Human habitation	Adult	1%	Field	External surfaces	[55]
*Eupenicillium*	*Eupenicillium spp.*	Medical	Worldwide	Human habitation	Adult	1%	Field	External surfaces	[55]
*Moniliella*	*Moniliella spp.*	Medical and veterinary	Worldwide	Human habitation	Adult	9%	Field	External surfaces	[55]
*Nigrospora*	*Nigrospora spp.*	Agricultural	Worldwide	Human habitation	Adult	1%	Field	External surfaces	[55]
*Rhizopus*	*Rhizopus spp.*	Veterinary	Worldwide	Human habitation	Adult	2%	Field	External surfaces	[55]
*Scopulariopsis*	*Scopulariopsis spp.*	Veterinary	Worldwide	Human habitation	Adult	2%	Field	External surfaces	[55]
*Mucorales*	*Mucorales spp.*	Medical	Worldwide	Hospitals	Adult	11%	Field	External surfaces	[59]
*Rhodotorula*	*Rhodotorula spp.*	Medical/veterinary	Worldwide	Hospitals	Adult	8.4%	Field	External surfaces	[59]
*Moniliella*	*M. suaveolans*	Medical	Worldwide	Human habitation	Adult	–	Field	External surfaces	[60]

**Table 3:** Parasites that have been isolated from house flies, including the site of isolation, the frequencies and their distribution

Parasite genera	Species	Medical or veterinary importance	Geographical occurrence	Site of specimen collection	Host stage infected	Prevalence	Lab or field study	Site of isolation	References
*Ascaris*	*A. lumbricoides*	Medical	Worldwide	Slaughter houses, dump sites, human habitation, open fields	Adult	12.6–14.29%	Field	External surfaces	[61–63, 98, 99]
	*A. suum*	Veterinary	Worldwide	piggery	Adult	62%	Field/lab	External surfaces/internal organs	[54]
*Entamoeba*	*E. histolytica*	Medical	Worldwide	Slaughter houses, dump sites, human habitation, open fields	Adult	35.43–53.57%	Field	External surfaces	[61–63]
Hookworm	*Ancylostoma duodenale/Necator americanus*	Medical	Worldwide	Slaughter houses, dump sites, human habitation, open fields	Adult	8.93%	Field	External surfaces	[61, 62, 64]
*Trichuris*	*T. trichiura*	Medical	Worldwide	Slaughter houses, dump sites, human habitation, open fields, piggery	Adult	12.5–74.0%	Field	External surfaces	[61–64]
	*T. suis*	Medical/veterinary	Worldwide	Piggery	Adult	–	Field/lab	External surfaces/internal organs	[54]
*Strongyloides*	*S. stercoralis*	Medical	Developing countries	Slaughter houses, dump sites, human habitation, open fields	Adult	10.7%	Field	External surfaces/internal organs	[61]
	*S. ransomi*	Veterinary	Tropical regions	Piggery	Adult	21%	Field	External surfaces	[54]
Metastrongylus	*M. spp*	Veterinary	Worldwide	Piggery	Adult	–	Field	External surfaces/internal organs	[54]
Haematopinus	*H. suis*	Veterinary	Worldwide	Piggery	Adult	–	Field	External surfaces/internal organs	[54]
*Crytosporidium*	*C. parvum*	Medical/ Veterinary	Worldwide	Laboratory experiment	Adult/larvae	–	Lab	Internal organs	[65]
*Giardia*	*G. lamblia*	Medical	Developing countries	Human habitation, refuse dumps, tomato/vegetable and soft drink shops	Adult	23.62%	Field	External surfaces	[3, 63]
*Enterobius*	*E. vermicularis*	Medical	Worldwide but more prevalent in developed world	Poultry	Adult	–	Field	External surfaces	[3]
*Taenia*	*T. spp.*	Medical/veterinary	Worldwide	Human habitation, refuse dumps, tomato/vegetable and soft drink shops	Adult	15.75%	Field	External surfaces	[63, 64]
*Hymenolepis*	*H. nana*	Medical	Worldwide	Human habitation, refuse dumps, tomato/vegetable and soft drink shops	Adult	5.51%	Field	Internal organs	[63]

Pathogens were isolated more frequently from the body surfaces of the flies as reflected from 44 studies (44.44%), followed by 33 studies (33.33%) reporting isolation from both the body surfaces and the gut, while 22 studies (22.22%) indicated isolation from the gut. Most studies reported isolation of pathogens from adult flies 91 (91.92%), followed by larvae 5 (5.05%) and from both the adults and the larvae 3 (3.03%).

The most frequent method used in the isolation of pathogens was standard cultural methods 77 (77.78%), followed by molecular methods (such as polymerase chain reaction [PCR] or sequencing) 14 (14.14%) and other parasitological techniques 8 (8.08%).

Among the bacterial pathogens isolated, 7 studies reported virulent bacteria (8.97%), 14 reported bacteria carrying genes which confer resistance to multiple antibiotics (17.95%), and the enteric bacteria were the most frequently isolated bacteria as shown in 55 studies (70.51%) (Table 1). Among the parasites, *Ascaris spp*. *Entamoeba spp*., hookworms and *Trichiuris spp*. were most frequently reported (Table 2). Among the fungi, *Penicillum spp*., *Aspergillus spp*., and *Candida spp*. were the most frequently reported (Table 3). Very few studies reported on viruses isolated from the house fly, most of which were experimental studies (Table 4).

**Table 4:** Viruses that have been isolated from house flies, including the site of isolation, the frequencies and their distribution

Virus family	Common name	Medical or veterinary importance	Geographical occurrence	Site of specimen collection	Host stage infected	Prevalence	Lab or field study	Site of isolation	References
Picornavirus	Senecavirus A	Medical/veterinary	Worldwide	Laboratory experiment	Adult	–	Lab	Internal organs	[65]
Filoviridae	Ebola virus	Medical	West and Central Africa	Laboratory experiment	Adult	–	Lab	Internal organs	[68]
Arteriviridae	Porcine reproductive and respirator syndrome virus	Veterinary	Worldwide	Piggery	Adult	–	Lab	Internal organs	[86]
Orthomyxoviridae	Avian Influenza virus H5N1	Veterinary	Worldwide	Laboratory experiment	Adult	–	Lab	Internal organs	[66]
Hytrosaviridae	Musca domestica salivary gland hypertrophy virus (MdSGHV)	Veterinary	Worldwide	Laboratory experiment	Adult	3–24%	Lab	Internal organs	[88]
Paramyxoviridae	Newcastle disease virus	Medical/veterinary	Worldwide	Laboratory experiment	Adult	–	Lab	Internal organs	[67]

## Discussion

This systematic review revealed a total of at least 130 pathogens that have been isolated from the house fly. Bacterial pathogens were by far the most frequently reported, suggesting the house fly may play an important role as vector of bacterial diseases. Fungi were the second most frequently isolated pathogens followed by parasites, and viruses were the least frequent. The differences in the rate of isolation of these pathogens could be attributed to individual biases at the level of the study, pertaining to the method used in the isolation of the pathogens. Most of the articles reviewed used standard cultural methods for the isolation of pathogens, which may have skewed the outcome towards bacterial pathogens; more advanced methods including cell culture and PCR, which are required for the detection of viruses, are expensive and not readily available. This may explain the low number of reports on isolation of viruses from house flies.

Pathogens were more frequently isolated from the body surfaces of house flies, especially from those captured from within human habitations and animal farms. House flies habitually feed on feces, animal manure, carrion and other decaying organic matter. In the process of feeding, pathogens stick on their mouth parts, wings, legs and other body surfaces, which they carry back to human habitations and animal farms, where they live and complete their lifecycle [6]. The constant movement of the house fly back and forth from feces (or other animal waste) to food and drinking water therefore places humans and animals at risk of infection. The frequent isolation of pathogens from the body surfaces of the flies makes it plausible that when house flies transmit pathogens, they only act as mechanical vectors [22–24, 26]. Unlike in biological transmission, there is no multiplication (amplification) of the pathogen in the host in mechanical transmission. However, the fly has been demonstrated to carry sufficient quantity of pathogens on its body surface, enough to cause an infection [27]. The quantity of pathogens present in the gut is usually higher than the quantity present on the body surfaces, suggesting that feces and vomitus may also serve as a major route of transmission of pathogens [28, 94].

Enteric bacteria were the most frequently isolated bacteria [2–24, 27, 29–35, 37–39]. This could be due to the fact that house flies feed mainly on feces and other animal waste, which is a rich source of enteric bacteria. Some of the bacteria isolated from house flies were highly virulent species including enteropathogenic strains such as enteroaggregative *E. coli* (EAEC), enterohaemorhagic *E. coli* (EHEC), enterotoxigenic *E. coli* (ETEC), and enteropathogenic *E. coli* (EPEC) [18, 29–34], *Vibrio cholera* and *Bacillus anthracis* that cause enteric diseases, cholera and anthrax respectively. Others including *Klebsiella spp*., *Pseudomonas*, *Staphylococci*, *Streptococci*, *Clostridium spp*. and *Enterococci* to name just a few, are also important causes of diseases in humans (including nosocomial infection). Furthermore, several studies reported bacteria that were resistant to multiple antibiotics including *E. coli* (20,35,36), *Klebsiella pneumoniae* [15, 47] and *Pseudomonas aeruginosa* [15, 19, 48]. Most of the antibiotic resistant bacteria were isolated from flies caught in and around hospital environments and animal farms (where there is an extensive use of antibiotics as growth promoters) [15, 17–20, 49, 50], suggesting that house flies may also play a role in the dissemination of antibiotic resistant bacteria to different environments [17].

Fungi species frequently isolated from the house fly belonged to the genera: *Candida*, *Aspergillus*, and *Penicillium* [7, 51–60]. Some of these genera (including *Candida* and *Aspergillus*) contain fungi species that are important human pathogens, but most others contain fungi species that are of veterinary (e.g. *Microsporum*, *Rhizopus*, *Scopularipsis* and *Rhodotorula*) and agricultural importance (e.g. *Curvalaria* and *Nigrospora*). Furthermore, genera *Epicoccum* contain fungi species which are important allergens. Some species of fungi that have been isolated from the house fly were resistant to multiple antifungals, example of which includes *Candida* [51]. Most of the fungi that have been isolated from the house fly were reportedly isolated from the outer cuticle of the insect and rarely from internal organs, feces or vomitus.

Very few studies reported the isolation of parasites from the house fly. Among these studies, almost all the parasites described were isolated from the body surfaces of the flies. The parasites species frequently reported belonged to the genera: *Ascaris*, *Entamoeba*, *Trichiuris*, and the hookworms [61–64]. These parasites commonly cause enteric diseases in humans and their frequent occurrence on the house fly could also be attributed to the food source of the house fly. Parasites of the genera *Metastrongylus* and *Heamatopinus*, which are known to be strict pathogens of domestic animals including pigs were also reported [54].

Reports of the isolation of viruses from wild-caught flies are very rare. However, house flies were reported to be capable of carrying a number of viruses in laboratory experiments. The majority of these viruses were of veterinary importance including the *Senecavirus A* whose natural hosts are pigs and cows [65]; the porcine reproductive and respiratory syndrome virus which causes a disease of pigs called porcine reproductive and respiratory syndrome (PRRS), also referred to as the blue-ear pig disease; Avian influenza virus and Newcastle disease virus which cause diseases in birds including poultry [66, 67]. In addition, one study demonstrates the ability of the house fly to carry the Ebola virus in laboratory experiments [68]. However, its role in the transmission of the virus is still to be confirmed.

### Study limitations

Although this systematic review addresses a key gap in the evidence base by identifying the types and prevalence of pathogens carried by the house fly, there are some key limitations in the evidence collected. Firstly, the survival of these pathogens on the house fly and the house fly’s role in the transmission of these pathogens to humans and animals remains largely undefined. Secondly, it is unclear how representative these pathogens reported are of the wider population of pathogens that are carried by the house fly.

### Future perspectives

Mechanical transmission of pathogens by arthropods including house flies is often overlooked because too much importance is given to biologically transmitted diseases such as malaria, yellow fever etc. [26]. Nevertheless, there is enough evidence to show that house flies can carry pathogens capable of causing serious diseases in humans and domestic animals, and should therefore be controlled. The control of the house fly can be achieved by physical (such as composting manure [95, 96]), chemical and biological methods. The use of chemical pesticides, which is the most common method today, is fast losing grounds due to the growing resistance by the house fly and other pests, couple to the effects they may have on non-target organisms [97–99], have led to the consideration of other methods, including biological control. Biological control agents including fungi of the genera *Metarhizium* and *Beauveria*, and bacteria including *Bacillus thuringiensis* can be used to control the housefly [93, 97]. Furthermore, the sequencing of the genome of the house fly presents new opportunities for the identification of novel targets for controlling the housefly and also for understanding the mechanism of resistance to insecticides as well as the genetic adaptation of the house fly to high pathogen loads [69].

## Conclusion

This review showed that the common house fly is a mechanical vector of a diverse range of pathogens including bacteria, fungi, viruses and parasites. However, more studies on identifying new pathogens and the survival of these pathogens are needed.

## Additional file

Additional file 1: PRISMA (Preferred Reporting Items for Systematic Reviews and Meta-Analyses) Checklist. (PDF 490 kb)

## Abbreviations

EAEC: Enteroaggregative *Escherichia coli*
EHEC: Enterohaemorhagic *Escherichia coli*
EPEC: Enteropathogenic *Escherichia coli*
ETEC: Enterotoxigenic *Escherichia coli*
PCR: Polymerase chain reaction
PRRS: Porcine reproductive and respiratory syndrome
